# Epigenetic Features of Human Perinatal Stem Cells Redefine Their Stemness Potential

**DOI:** 10.3390/cells9051304

**Published:** 2020-05-24

**Authors:** Giulia Gaggi, Andrea Di Credico, Pascal Izzicupo, Ivana Antonucci, Clara Crescioli, Viviana Di Giacomo, Annalisa Di Ruscio, Giovanni Amabile, Francesco Alviano, Angela Di Baldassarre, Barbara Ghinassi

**Affiliations:** 1 Department of Medicine and Aging Sciences, “G. D’Annunzio” University of Chieti- Pescara, 66100 Chieti, Italy; giulia.gaggi@unich.it (G.G.); andrea.dicredico@unich.it (A.D.C.); izzicupo@unich.it (P.I.); 2 Department of Psychological, Humanities and Territorial Sciences, “G. D’Annunzio” University of Chieti, Pescara, 66100 Chieti, Italy; i.antonucci@unich.it; 3 Department of Movement, Human and Health Sciences, University of Rome “Foro Italico”, 00135 Rome, Italy; clara.crescioli@uniroma4.it; 4 Department of Farmacy, “G. D’Annunzio” University of Chieti- Pescara, 66100 Chieti, Italy; viviana.digiacomo@unich.it; 5 Department of Translational Medicine, University of Eastern Piedmont, 28100 Novara, Italy; adirusci@bidmc.harvard.edu; 6 Beth Israel Deaconess Medical Center, Harvard Medical School Initiative for RNA Medicine, Harvard Medical School, Boston, MA 02115, USA; 7 Enthera Srl, 20123 Milan, Italy; giovanni.amabile@entherapharmaceuticals.com; 8 Department of Experimental, Diagnostic and Specialty Medicine, Unit of Histology, Embryology and Applied Biology, University of Bologna, 40126 Bologna, Italy; francesco.alviano@unibo.it

**Keywords:** perinatal stem cells, amniotic fluid stem cells, amniotic epithelial cells, fetal membrane mesenchymal stromal cells, NANOG, OCT4, SOX2, DNA methylation, miRNAs expression, telomere length

## Abstract

Human perinatal stem cells (SCs) can be isolated from fetal annexes without ethical or safety limitations. They are generally considered multipotent; nevertheless, their biological characteristics are still not fully understood. The aim of this study was to investigate the pluripotency potential of human perinatal SCs as compared to human induced pluripotent stem cells (hiPSCs). Despite the low expression of the pluripotent factors NANOG, OCT4, SOX2, and C-KIT in perinatal SC, we observed minor differences in the promoters DNA-methylation profile of these genes with respect to hiPSCs; we also demonstrated that in perinatal SCs miR-145-5p had an inverse trend in comparison to these stemness markers, suggesting that NANOG, OCT4, and SOX2 were regulated at the post-transcriptional level. The reduced expression of stemness markers was also associated with shorter telomere lengths and shift of the oxidative metabolism between hiPSCs and fetal annex-derived cells. Our findings indicate the differentiation ability of perinatal SCs might not be restricted to the mesenchymal lineage due to an epigenetic barrier, but other regulatory mechanisms such as telomere shortening or metabolic changes might impair their differentiation potential and challenge their clinical application.

## 1. Introduction

Stem cells (SCs) are undifferentiated and unspecialized cells that have the ability to self-renew and differentiate into specialized cells through asymmetric and symmetric divisions [1]. Human (h) SCs are generally divided into four main groups: embryonic SCs (hESCs), induced pluripotent SCs (hiPSCs), perinatal SCs, and adult or somatic SCs. Adult SCs reside in specialized niches within organs during post-natal life; they usually are oligo- or unipotent and are critical for tissue regeneration [2]. hESCs are pluripotent cells that can be isolated from the inner cell mass of the blastocyst; they can differentiate into all embryonic and extra-embryonic tissues [3]; however, their use is impaired by ethical and clinical concerns as their isolation causes the destruction of a blastocyst and they can form teratomas [4]. These ethical problems were overcome when Yamanaka et al. generated hiPSCs from adult human fibroblasts through the retroviral transduction of *OCT3/4, SOX2, KLF4*, and *c-MYC*: due to the activity of these factors, differentiated cells can be reprogrammed and returned to a pluripotent state. hiPSCs have a self-renewal capacity, thus representing an unlimited source for research and are quite similar to hESCs in terms of their transcription programs and global chromatin configuration [5]. Despite the high hopes generated by hiPSCs research, the biology of these cells still casts shadows on their clinical application. Technical hurdles in the reprogramming result in a different quality of hiPSCs, the epigenetic memory influences the differentiation efficiency and their biological instability is responsible for poorly controlled risks of unpredictable reactions in patients [6].

Currently, only a few clinical trials have tested the safety of hiPSCs for therapeutic purposes [7]. For this reason, scientists have focused their attention on SCs that can be isolated from human perinatal tissue. Placenta and amniotic fluid represent an interesting source of SCs for clinical and research purposes, as they are easily obtainable and their use does not raise ethical concerns; moreover, they display paracrine activity by secreting cytokines that are involved in angiogenesis, immune modulation, and tissue repair (for a review, see Gaggi et al. [4]). They are generally considered multipotent, nevertheless, their real position in the stemness hierarchy and their differentiative potential remain unclear. Different cell populations can be isolated from fetal annexes.

Human fetal membrane mesenchymal stromal cells (hFM-MSCs) can be isolated from the amniochorionic membrane. They have a fibroblast-like shape and can be expanded in vitro for at least nine passages without changes to the cell morphology [8]. hFM-MSCs can differentiate into mesodermal lineages and have a low immunogenicity, expressing at a very low level the HLA class I and class II antigens on their surface and lack co-stimulatory molecules, such as CD80, CD40, and CD40 ligands [8].

Human amniotic epithelial cells (hAECs), derived from the amniotic membrane, originate from the epiblast at days 8–9 during the development, before gastrulation [9]. They express some markers of pluripotency; however, unlike ESCs, they neither cause teratomas nor activate the immune system after transplantation in vivo. In adhesion, hAECs show the cuboidal morphology of epithelial cells; whereas, in suspension culture, they form an embryoid body structure, a characteristic typical of ESCs [4,9]; however, in vitro they have a low proliferation efficiency, and their survival is limited to five to six passages.

SCs have been also isolated from the amniotic fluid. Human amniotic fluid SCs (hAFSC) can be easily recovered after amniocentesis and are able to differentiate into several cell lineages [4,10]. They are not tumorigenic; thus, they represent a good potential candidate for therapeutic applications [11].

Although some features of perinatal SCs support their potential use in regenerative medicine, a more extensive analysis of their biological characteristics is needed prior to encouraging the translation of the preclinical studies into therapy. The aim of this study was to better delineate the stemness features of human perinatal SCs by outlining some of their epigenetic, genetic, and biological characteristics in comparison with hiPSCs.

## 2. Materials and Methods

### 2.1. Culture of Human Cells

All culture mediums and supplements were purchased by Thermo Fisher Scientific (Waltham, MA, USA), unless otherwise indicated.

The hiPSCs [12] were cultured on a monolayer of irradiated-murine fibroblasts (Thermo Fischer Scientific, Waltham, MA, USA) in Dulbecco’s Modified Eagle’s Medium/Nutrient Mixture F-12 (DMEM/F12) supplemented with 20% KnockOut Serum Replacement (KO), 1% penicillin/streptomycin, 2 mM l-glutamine, 5 ng/mL basic fibroblast growth factor (bFGF), and 1% MEM nonessential amino acid.

Placenta and amniotic fluid samples were obtained after written informed consent in accordance with the Declaration of Helsinki. All samples had normal diploid karyotypes. The study was approved by the ethics committees of the “G. d’Annunzio” University of Chieti-Pescara, ASL Lanciano-Chieti-Vasto and of St. Orsola-Malpighi University Hospital, Italy and all experiments were performed in accordance with relevant guidelines and regulations. The hAFSCs were isolated as previously described [13] from 15 women undergoing amniocentesis for prenatal diagnosis at 16–17 weeks of pregnancy; cells were cultured in Iscove’s modified Dulbecco’s medium (IMDM) supplemented with 20% fetal bovine serum (FBS), 1% penicillin/streptomycin, 2 mM l-glutamine, and 5 ng/mL bFGF. The hFM-MSCs and hAECs were isolated from the term placentas of 6 healthy donors mothers as previously described [14]. Cells were cultured in DMEM 10% FBS, 1% penicillin/streptomycin, and 2 mM l-glutamine. For hAECs culture, medium was supplemented with 10 ng/mL epidermal growth factor (Sigma-Aldrich, Saint Louis, MO, USA). All the cell culture media and supplements are summarized in Table 1.

All the experiments were performed at early cell passages (2–4 passages).

### 2.2. RNA Extraction and Reverse Transcription

RNA from hESCs was purchased from Celprogen (Torrance, CA, USA). The hiPSCs, hAFSCs, hFM-MSCs, and hAECs were lysed with QIAzol lysis reagent (QIAGEN, Hilden, Germany). The total RNA was extracted using the miRNeasy Mini Kit (QIAGEN, Hilden, Germany) according to the manufacturer’s procedure [15]. For reverse transcription, 1 μg of RNA was retrotranscribed by the high-capacity cDNA reverse transcription kit (Thermo Fisher Scientific, Waltham, MA, USA) according to the manufacturer’s procedure. For the reverse transcription of miRNAs, 1 μg of RNA was retrotranscribed using the high-capacity cDNA reverse transcription kit (Thermo Fisher Scientific, Waltham, MA, USA) following the stem and loop reverse transcription (RT) protocol [16]: buffer RT 10× 2 μL, dNTPs 0.8 μL, U6 RT primer (10 pmol/μL) 4 μL, miR-299-3p RT primer (10 pmol/μL) or miR-150-5p RT primer (10 pmol/μL) 4 μL, or miR-145-5p RT primer (10 pmol/μL), MultiScribe™ 2 μL, and H_2_O 15 μL.The primers used for the experiments are summarized in Table 2.

### 2.3. Real Time Quantitative PCR (qPCR)

For all the examined mRNAs, qPCR analysis was performed using SYBR green (PowerUp SYBR Green Master mix, Thermo Fisher Scientific, Waltham, MA, USA) in a final volume of 20 µL. The samples were analyzed in triplicate in MicroAmp optical 96-well reaction plates (Thermo Fisher Scientific, Waltham, MA, USA). Since normalization to GAPDH has been shown to be not very accurate [17], the more stable *18S* was used as a housekeeping gene. Two different mixes were initially prepared to be subsequently distributed with a 1:1 ratio in each well. We mixed SYBR green-primers (10 µL/well): SYBR green 9.2 µL, mixed primers FW + RV (10 pmol/µL) 0.8 µL. Then mixed cDNA-RNase free water (10 µL/well): cDNA (12.5 ng/well) 2 µL, and RNase free water 8 µL. The samples were run as follows: step 1, 95 °C for 10 min; step 2, 95 °C for 15 s; and step 3, 60 °C for 1 min. Steps 2 and 3 were repeated for 40 cycles. The authenticity of the PCR products was verified by a melt-curve analysis. For data analysis, the Ct value of all genes analyzed was normalized to the Ct of the housekeeping gene *18S*, resulting in the ΔCt value. Next, the ΔΔCt value was obtained subtracting the ΔCt value of each experimental condition from ΔCt value of the control condition (hESC). Finally, the fold change was generated using the formula 2^−ΔΔCt^.

To detect the miR-299-3p, miR-150-5p, and miR-145-5p, qPCR was performed as previously described, but with using *U6* as a housekeeping gene and 25 ng of cDNA per reaction. For data analysis, the ΔΔCt method was used with hiPSCs as a control condition. The primers used are listed in Table 3.

### 2.4. MTT (3[4,5-Dimethylthiazol-2yl]-2,5-Diphenyl Tetrazolium Bromide) Assay

The cell metabolic activity was measured using a MTT (3[4,5-dimethylthiazol-2yl]-2,5-diphenyl tetrazolium bromide) growth assay (Sigma-Aldrich, Saint Louis, MO, USA). The cells were seeded at 15.625 cell/cm^2^ and after 24 h treated with 0.5 mg/mL MTT for 4 h. After incubation, formazan, generated by MTT reduction, was solubilized in dimethyl sulfoxide (DMSO) for 30 min at 37 °C. The absorbance of each well was detected at 540 nm.

### 2.5. Flow Cytometry

For flow cytometry, the cells were treated with the FIX and PERM^®^ Kit (Thermo Fisher Scientific, Waltham, MA, USA) and then incubated for 1 h at room temperature with anti-Oct-4 Alexa Fluor 488 conjugated (Cell Signaling, Danvers, MA, USA) 1:50, anti-Nanog Alexa Fluor 647 conjugated (Becton Dickinson, Franklin Lakes, NJ, USA) 1:20, anti-c-Kit PE conjugated (Becton Dickinson, Franklin Lakes, NJ, USA) 1:5, anti-Sox2 Alexa Fluor 488 conjugated (Becton Dickinson, Franklin Lakes, NJ, USA) 1:5 or appropriate isotype controls [20] (all from Becton Dickinson, NJ, USA). Cytometric analyses were performed with a Cytoflex cytometer (Beckman Coulter, Brea, CA, USA), and the data were analyzed with CytExpert Acquisition and Analysis Software (Beckman Coulter, Brea, CA, USA).

### 2.6. Immunofluorescent Analysis

For the immunofluorescence, the cells were treated as previously reported [21,22]. The samples were incubated with anti-Oct-4 Alexa Fluor 488 conjugated (Cell Signaling, Danvers, MA, USA) 1:50, anti-Nanog Alexa Fluor 488 conjugated (Becton Dickinson, Franklin Lakes, NJ, USA) 1:5, anti-c-Kit PE conjugated (Becton Dickinson, Franklin Lakes, NJ, USA) 1:5, and anti-Sox2 Alexa Fluor 488 conjugated (Becton Dickinson, Franklin Lakes, NJ, USA) 1:5. The nuclei were stained with DAPI (Thermo Fisher Scientific, Waltham MA, USA). The images were acquired with Camera Axiocam 503 Mono and analyzed with ZEN Software (Carl Zeiss, Jena, Germany).

### 2.7. Pyrosequencing

Genomic DNA was extracted from hiPSCs, hMFs, hAECs, and hAFSCs using the blood and cell culture mini kit (QIAGEN, Hilden, Germany) and quantified by Qubit 4 (Thermo Fisher Scientific, Waltham, MA, USA) according to the manufacturer’s instructions. We used 100 ng of genomic DNA in the bisulfite conversion reactions using the EZ DNA methylation direct kit (Zymo Research, Irvine, CA, USA) according to the manufacturer’s instructions. The bisulfite-treated samples were then amplified by PCR (SimpliAmp, ThermoFisher Scientific, Waltham, MA, USA), using forward and reverse primers (see Table 3 for PCR primers and conditions), with one primer biotinylated. The bisulfite-converted DNA was amplified with the KAPA HiFi HotStart Uracil + Ready Mix PCR kit (Kapa Biosystems, Roche, Basilea, Switzerland). The amplification cycles included: denaturation (at 95 °C for 3 min), 30 cycles of denaturation (98 °C for 20 s), annealing (15 s; annealing temperatures are reported in Table 4), and extension (72 °C for 1 min), followed by 1 cycle of final extension (72 °C for 1 min). The PCR products were bound to Streptavidin Sepharose HP (Diatech phrarmacogenetics, Jesi, Italy), and captured using the PyroMark^®^ Q96 vacuum preparation tool (Qiagen, Hilden, Germany). After washes, the biotinylated single stranded PCR products were then released into a 96-well format plate (Pyro ID plate, Diatech pharmacogenetics, Jesi, Italy) containing 2 µL of 100 pmol pyrosequencing primer suspended in 38 µL of annealing buffer. The plate was loaded into the PyroMark^®^ Q96 instrument (Qiagen, Hilden, Germany) and analyzed using the PyroMark^®^ Q96 software for CpG methylation quantitation. The percentage of methylation was expressed for each DNA locus as %5-mC divided by the sum of methylated and unmethylated cytosines.

### 2.8. Absolute Telomere Length Quantification

DNA was extracted from hiPSCs, hFM-MSCs, hAECs, and hAFSCs using the blood and cell culture mini kit (QIAGEN, Hilden, Germany), quantified by Qubit 4 (Thermo Fisher Scientific, Waltham, MA, USA) and then analyzed by the absolute human telomere length quantification qPCR assay kit (ScienCell, Carlsbad, CA, USA) according to the manufacturer’s instructions. The telomere length per each chromosome end (in kb) was calculated using a reference genomic DNA sample with a telomere length per each chromosome end of 7.70 ± 0.57 kb. To calculate the telomere length for each sample, a reference genomic DNA sample was used.

### 2.9. Statistical Analysis

All data are presented as the mean ± SD of 5 independent experiments. A statistical analysis was performed using the one-way analysis of variance (ANOVA) and Tukey’s post-hoc analysis. The level of significance was set at *p* < 0.05.

## 3. Results

### 3.1. Promoter DNA Methylation Profile of Stemness Gene in Perinatal SCs

During the differentiation process, DNA methylation plays an important role in silencing pluripotency-related genes by inhibiting the binding of transcription factors or by recruiting proteins involved in gene repression [23]. It is well known that NANOG, OCT4, and SOX2 represent the triad of master transcription factors of pluripotency [24]. For this reason, we investigated the DNA methylation profile of these transcription factors and C-KIT, another stemness marker expressed in ESCs and other types of SCs [25,26,27]. The analysis of the methylation levels was carried out by the pyrosequencing of CpGs within the promoters of *NANOG*, *OCT4*, *SOX2,* and *C-KIT*.

**Figure 1:**
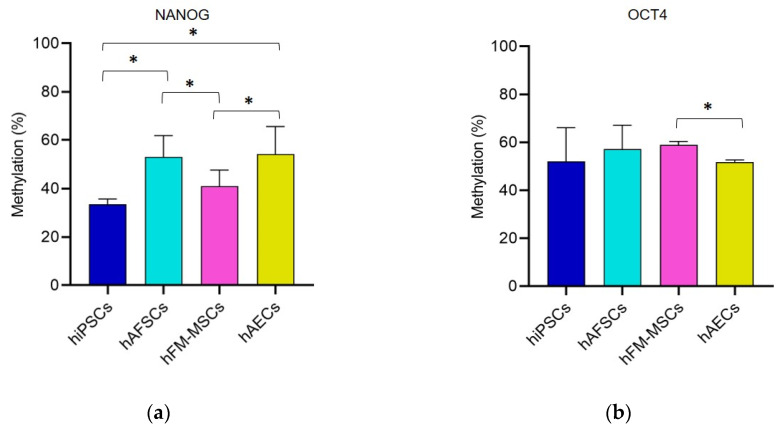

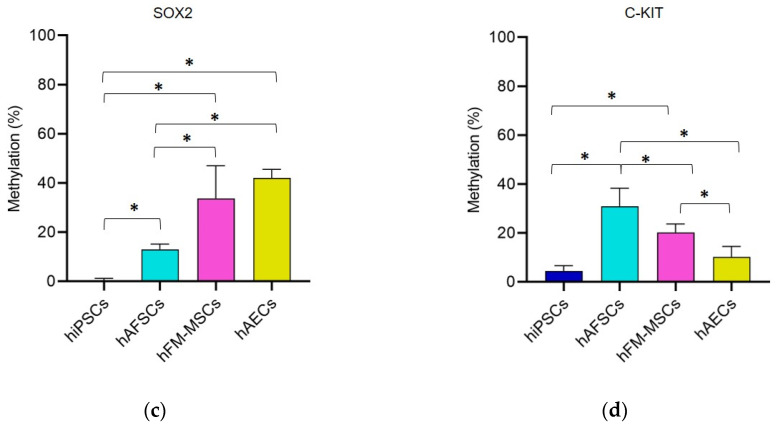
The methylation profile of *NANOG, OCT4*, *SOX2*, and *C-KIT* in hiPSCs and perinatal stem cells (SCs). Pyrosequencing results showed the percentage of methylation levels in the promoter region of (**a**) *NANOG*, (**b**) *OCT4*, (**c**) *SOX2*, and (**d**) *C-KIT* in SCs from different sources. The graphs show the mean ± SD of 5 independent experiments, * *p* < 0.05.

Due to the legal issues related to the manipulation of hESCs in our country, we used hiPSCs as a positive control for our study, knowing that the DNA methylation levels at the promoter of these pluripotent factors is low or undetectable [28,29]. For *NANOG*, six CpGs were studied: the results indicated that the *NANOG* methylation levels were comparable between hiPSCs and hFM-MSCs, while they were higher relative to the hiPSCs in hAFSCs and hAEC (Figure 1a).

The pyrosequencing of ten CpGs inside the *OCT4* promoter did not demonstrate significant differences in the methylation status between hiPSCs and the SCs isolated from the different placental parts. Among the perinatal SCs, the percentage of *OCT4* methylation in hAECs was significantly lower than in hFM-MSCs (Figure 1b).

The investigation of six CpGs into the *SOX2* promoter highlighted an important hypomethylation for hiPSCs in comparison with hAFSCs, hAECs and hFM-MSC. Among the perinatal SCs, hAFSC showed significantly lower *SOX2* methylation levels than hFM-MSCs and hAECs (Figure 1c).

Finally, pyrosequencing of eight CpGs inside the *c-KIT* promoter displayed a significant lower percentage of DNA methylation in hiPSCs than in fetal annex-derived SCs. Specifically, hAFSCs were characterized by the highest methylation level, while the hAECs by the lowest (Figure 1d).

These data demonstrate that the master genes of pluripotency are hypomethylated in hiPSCs when compared to perinatal SCs. Remarkably the percentage of the *OCT4* DNA methylation promoter was similar among all the different SC populations analyzed, and even in *NANOG* the levels of DNA methylation were very similar between hiPSCs and hFM-MSCs; in addition, perinatal SCs isolated from fetal cells showed a different epigenetic profile with diverse methylation statuses of the stemness marker genes.

### 3.2. Analysis of Pluripotency Gene and Protein Expression in hiPSCs and Human Perinatal SCs

**Figure 2:**
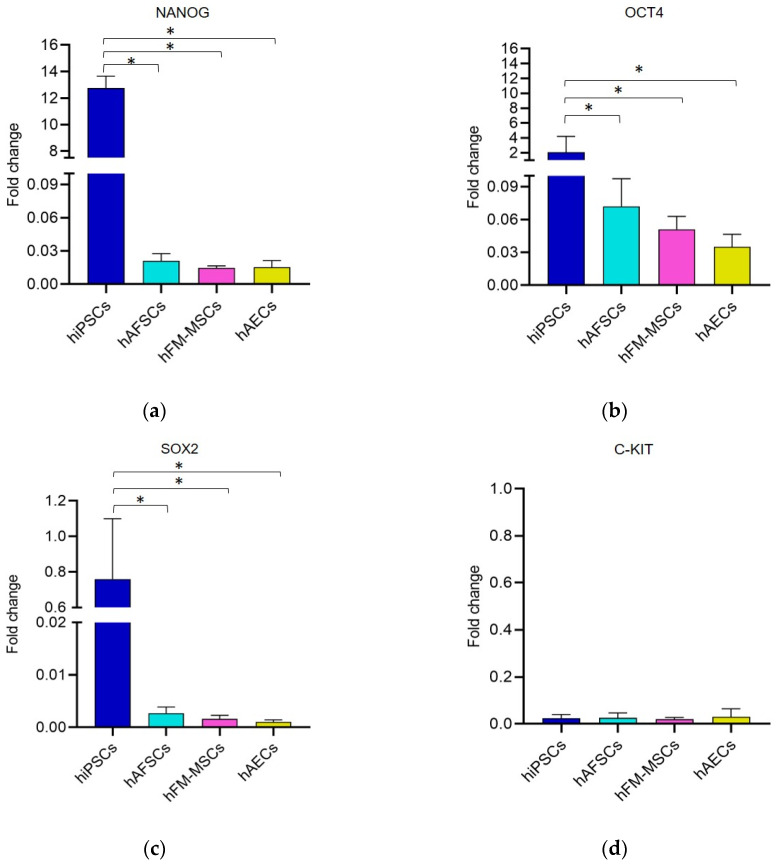
The gene expression analysis for “stemness genes” in hiPSCs and perinatal SCs. The gene expression of (**a**) *NANOG*, (**b**) *OCT4*, (**c**) *SOX2*, and (**d**) *C-KIT* detected by real time PCR in SCs from different sources. The fold changes were determined from the −ΔΔCt values calculated using *18S* as a reference gene and normalized to hESCs, as the control condition. The graphs show the mean ± SD of 5 independent experiments, * *p* < 0.05.

To assess whether the changes in the DNA methylation profile mirrored the expression of the pluripotent factors we assessed gene expression of *NANOG, OCT4, SOX2,* and *C-KIT* by performing quantitative real time PCR on RNA obtained from the different SCs populations. Unexpectedly, perinatal SCs displayed significantly lower mRNA levels of pluripotency markers than hiPSCs, with the exception of *C-KIT*, that was barely detectable in all the tested cells (Figure 2).

**Figure 3:**
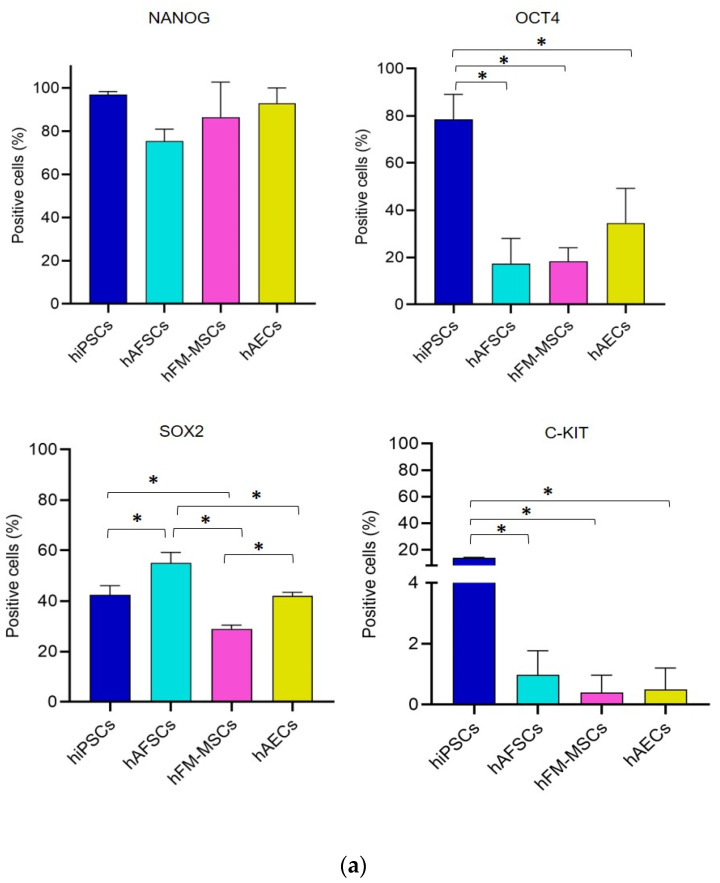

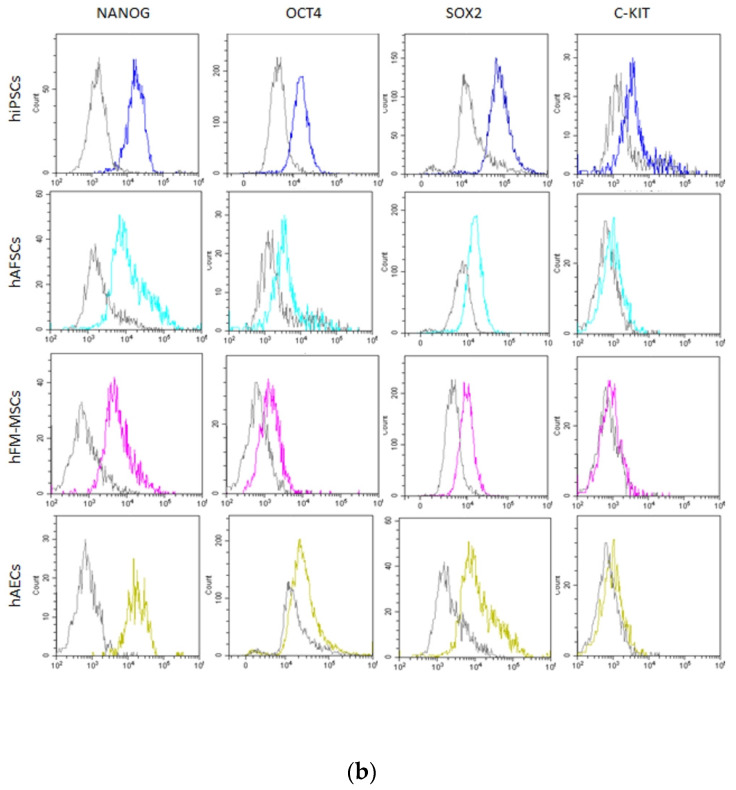
Protein expression of NANOG, OCT4, SOX2, and C-KIT by a flow cytometry analysis in hiPSCs and perinatal SCs. (**a**) Graph bars show the percentage of NANOG, OCT4, SOX2, and C-KIT positive cells in hiPSCs and perinatal SCs, obtained by flow cytometry. The graphs show the mean ± SD of 5 independent experiments, * *p* < 0.05. (**b**) Representative flow cytometry histograms of NANOG, OCT4, SOX2, and C-KIT in hiPSCs and perinatal SCs, as indicated. Grey histograms represent the isotype controls.

**Figure 4:**
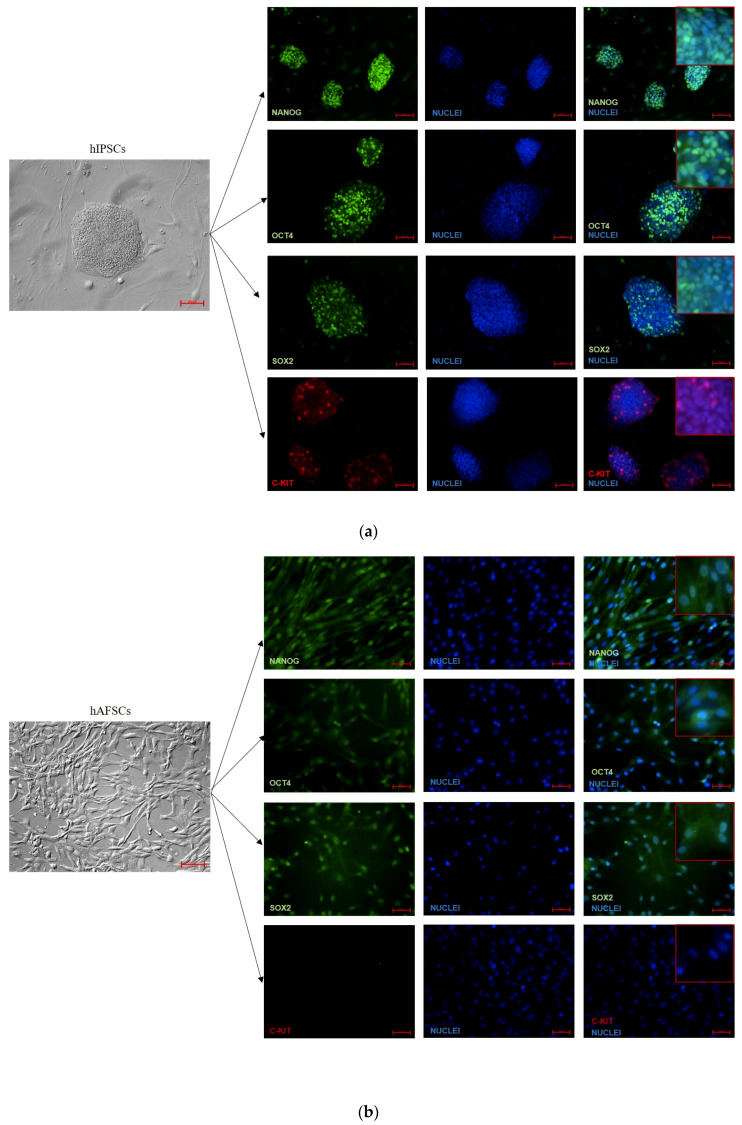

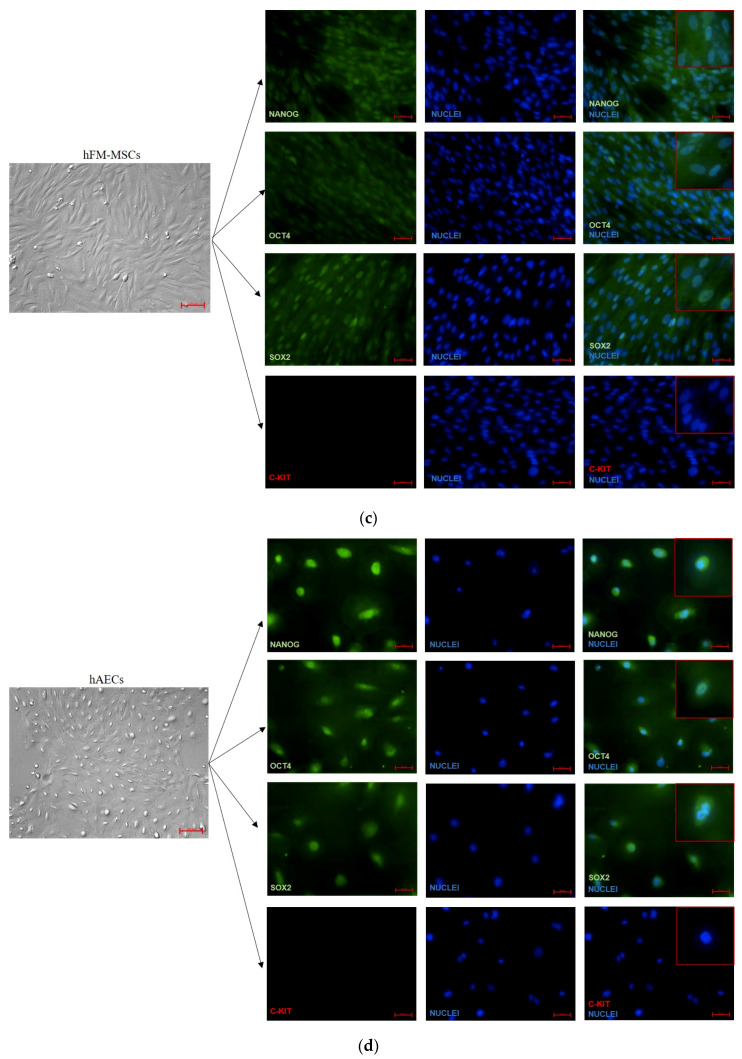
Immunofluorescent analysis for the NANOG, OCT4, SOX2, and C-KIT expression in hiPSCs and perinatal SCs. NANOG, OCT4, SOX2 (green fluorescence), and C-KIT (red fluorescence) detection in (**a**) hiPSCs, (**b**) human amniotic fluid SCs (hAFSCs), (**c**) fetal membrane mesenchymal stromal cells (hFM-MSCs), and (**d**) human amniotic epithelial cells (hAECs). The nuclei were counterstained with DAPI (blue). Brightfield images (in grey) highlight the different morphology of the cell populations. The cells were observed with AxioVert A1 using plasDIC-plan neofluar. Original Magnification: brightfield: 10×; Fluorescence: 20×; inset: 40×. Scale bars: brightfield images: 100 μm; fluorescence images: 50 μm. The images are representative of 5 independent experiments.

We then completed the analysis of the pluripotency markers by analyzing their protein expression and localization by means of cytometric and immunofluorescence analyses. The data are shown in Figure 3 and Figure 4. Despite a comparable percentage of NANOG^+^ cells in both hiPSCs and fetal annex-derived SCs, the protein expression measured by the mean fluorescence intensity (MFI) deeply varied from very high (in hiPSCs) to low (in hFM-MSCs) levels. The immunofluorescence analysis confirmed this heterogeneity in the expression levels: while NANOG in the hiPSCs displayed mainly a nuclear localization, as demonstrated by the strong corresponding staining, and suggesting an active state of this transcription factor; hAECs showed weaker immunoreactivity, still maintaining the nuclear distribution, contrariwise a fainter fluorescence at both the nuclear and cytoplasmic compartments was observed in hAFSCs and hFM-MSCs.

As expected OCT4 displayed higher levels of both positive cells and MFI values in hiPSCs. Differences in the OCT4 expression levels among the perinatal SCs were also demonstrated. These data were confirmed by the immunofluorescence: as for NANOG, OCT4 showed a stronger nuclear staining in hiPSCs and hAECs, whereas hAFSCs and hFM-MSCs were characterized by a weaker positivity distributed both in the cytoplasmatic and nuclear compartments.

The cytometric analysis for SOX2 evidenced significant differences among the different SCs populations. The immunofluorescence analysis demonstrated a strong nuclear localization in hiPSCs, hAFSCs, and hAECs, whereas in hFM-MSCs the fluorescence clearly marked the cytoplasm. On the contrary, C-KIT was weakly expressed only by a low percentage of hiPSCs (13.7% ± 0.4%), being almost undetectable in hAFSCs, hFM-MSCs, and in hAECs.

Our results indicate that the gene and protein expression were generally lower in perinatal SCs in comparison to hiPSCs, even though the NANOG protein expression levels were comparable between hiPSCs, hAFSCs, and hAECs.

### 3.3. Perinatal SCs Expressed miRNAs That Are Involved in the Regulation of the “Stemness” Gene

We showed that a similar epigenetic profile between hiPSCs and perinatal SCs is in striking contrast to transcriptional and protein levels of the respective factors. Thus, we investigated the potential involvement of post-transcriptional microRNA-mediated mechanisms to explain this apparent discrepancy. Expression levels of human SCs miR-145-5p that downregulates the overall triad of the pluripotent factors, miR-150-5p and miR-299-3p, known to affect NANOG and OCT4 expression respectively [30,31,32,33] were assessed in human perinatal SCs and compared to those in hiPSCs.

**Figure 5:**
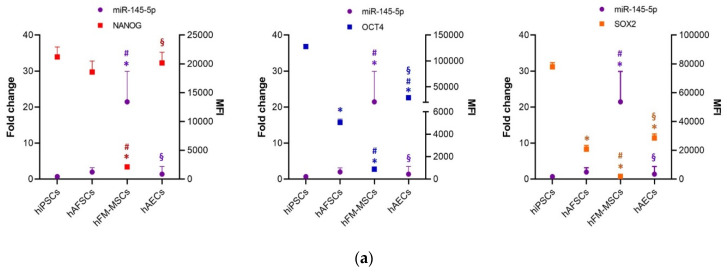

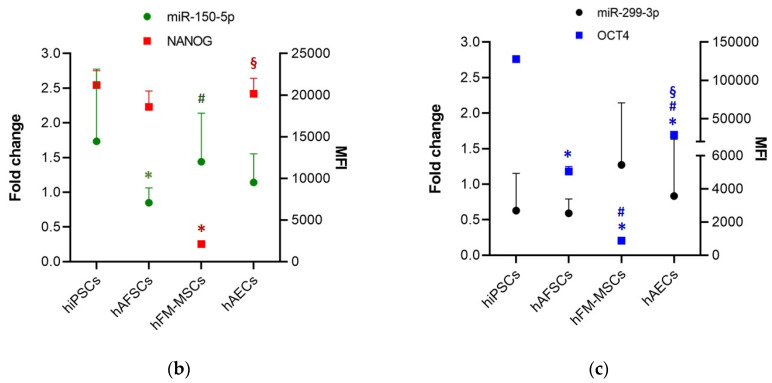
The detection of miR-299-3p, miR-150-5p, and miR-145-5p in hiPSCs and perinatal SCs and their relationship with the protein expression of NANOG, OCT4, and SOX2. The graph shows the (**a**) miR-145-5p expression, (**b**) miR-150-5p, and (**c**) miR-299-3p (primary y axis) and the expression of the NANOG, OCT4, and SOX2 proteins, as measured with the mean fluorescence intensity by the immunocytometry analysis (secondary y axis) in SCs from different fetal annexes. The miRNAs were detected by real time PCR, the fold change was determined from the −ΔΔCt values, calculated using *U6* as reference gene and normalized to hiPSCs, as the control condition. The graphs show the mean ± SD of 5 independent experiments, *p* < 0.05 with hiPSCs (*), hAFSCs (#), and hFM-MSCs (§).

Our analyses revealed that among the different SCs populations, the hFM-MSCs expressed significantly higher levels of miR-145-5p (fold change 21.4 ± 8.5) compared to hAFSCs and hAECs (fold change 1.96 ± 1.18 and 1.35 ± 2.15, respectively). The expression of NANOG, OCT4, and SOX2, measured as the MFI of the cytometric analysis, displayed an opposite trend to that of miR-145-5p, with hFM-MSCs characterized by the lowest and hiPSCs by the highest levels of protein expression of these stemness markers (Figure 5a).

hAFSCs displayed significantly lower levels of miR-150-5p with respect to hiPSCs, while hFM-MSCs and hAECs expressed comparable levels of this miRNA (Figure 5b). No relationship was observed in the trend of the miR-150-5p expression and the NANOG protein; however, hFM-MSCs showed significant differences of both miR-150-5p and NANOG profiles from hAFSCs.

Finally, the miR-299-3p expression level was compared between hiPSCs and perinatal SCs; only a trend of inverse correlation without reaching the statistical significance between the miRNA and OCT4 expression was observed (Figure 5c).

These results suggest that miRNAs, in particular miR-145-5p, may contribute to the different expression profiles of pluripotency triad proteins in perinatal SCs.

### 3.4. Telomere Length Quantification in hiPSCs and Perinatal SCs

**Figure 6:**
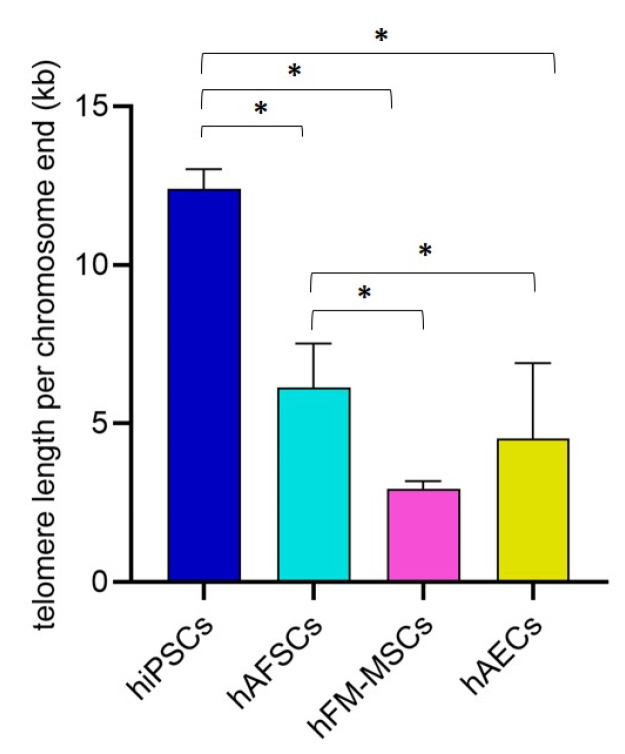
The telomere length per each chromosome end (in kb) in hiPSCs and perinatal SCs was quantified by qPCR. All the cells were at 2–4 passages. The graphs show the mean ± SD of 5 independent experiments, * *p* < 0.05.

In embryonic SCs, telomerase is activated maintaining the telomere length and cellular immortality; however, the level of telomerase activity seems to be low in the majority of SCs. As it is commonly known that telomeres shorten with aging, thus limiting the proliferative capacity of adult SCs, we performed an absolute quantification of telomere length in perinatal SCs at the second or third passage in comparison with hiPSCs. These cells have been suggested to have telomere lengths that are significantly longer than the parental differentiated cells, reaching a length comparable to ESCs [34]. Our data demonstrated that telomere length is statistically higher in hiPSCs as compared to hAFSCs, hFM-MSCs, and hAECs (12 ± 0.61 kb vs. 6.1 ± 1.4 kb, 2.9 ± 0.25 kb, and 3.2 ± 0.38 kb, respectively). Interestingly, hAFSCs displayed longer telomeres than hFM-MSCs and hAECs suggesting a higher stemness potential. No differences were found between hFM-MSCs and hAECs (Figure 6).

### 3.5. Metabolic Activity in hiPSCs and Perinatal SCs

It is well known that the cellular metabolism describes a defined biochemical phenotype of the cells and that it plays a pivotal role in the maintenance of stemness and generation of differentiated progenies [35,36].

**Figure 7:**
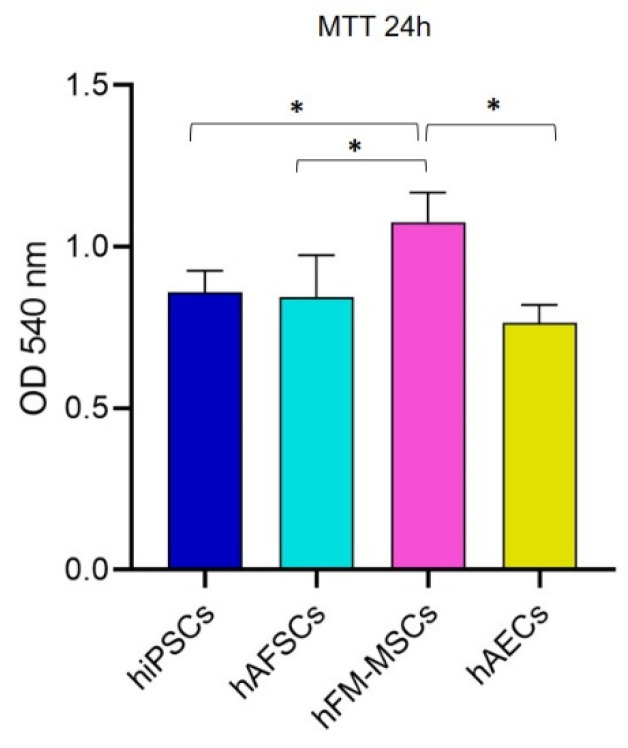
MTT assay for hiPSCs, hAFSCs, hFM-MSCs, and hAECs. The cells were seeded at 15.625 cell/cm^2^ and after 24 h they were treated with 0.5 mg/mL MTT for 4 h. The absorbance is expressed as measure of cell viability and their basal metabolism. The graphs show the mean ± SD of 5 independent experiments, * *p* < 0.05.

Thus, to complete the characterization of the perinatal SCs, we assessed the metabolic activity of the hiPSCs and perinatal SCs by an MTT assay 24 h after the seeding. The test showed that the metabolism of hAFSCs and hAECs was comparable to the hiPSCs one, while the hFM-MSCs were significantly even more metabolically active than the other perinatal SCs and hiPSCs (Figure 7).

## 4. Discussion

In the field of regenerative medicine, the highest differentiation efficiency is obtained from ESCs and iPSCs, but their use in clinical practice is controversial, as they can be tumorigenic. Moreover, the use of ESCs has also ethical limitations as their isolation involves the destruction of a blastocyst [37]. On the other hand, the technical hurdles in reprogramming can affect the quality and the efficiency of iPSC generation; thus, reprogrammed cells can have poorly controlled, unpredictable reactions during clinical applications [6]. Perinatal SCs can be easily isolated during routine medical procedures (amniocentesis) or from fetal annexes after delivery. Evidence suggests that these cells are not tumorigenic and have a low immunogenicity [4]. These properties make AFSCs, FM-MSCs, and AECs a good alternative to ESC and iPSCs in regenerative medicine applications.

SCs from human perinatal tissue cannot divide indefinitely in vitro and are generally considered to be multipotent as they are able to differentiate into various subtypes of the mesodermal lineage. Nevertheless, their biological characteristics and their real position in the stemness hierarchy is still unclear. We have already reported, indeed, that hAFSCs expressed mRNAs of pluripotent markers including *NANOG, OCT4, SOX2, FRAGILIS*, and *KLF4* [13]; moreover it was demonstrated that hAECs express markers typical of glial and neuron cells [38] and can differentiate into cells belonging to different germinal layers, such as cardiac, pancreatic, and hepatic-like cells [4,10]. Despite these results, no definitive experiments have been provided for the analysis of “stemness features” of different populations of perinatal SCs.

In our study we attempted to delineate some biological characteristics of the SCs derived from the different parts of the fetal annexes. The main findings of this study are that: *i.* Fetal annex-derived SCs expressed detectable levels of pluripotency markers. *ii.* Although they showed distinct protein levels, the *NANOG*, *OCT4,* and *SOX2* genes displayed a similar epigenetic profile in perinatal SCs and hiPSCs. *iii.* Their downregulation in placenta-derived SCs may be at least in part operated at the post-transcriptional level by specific miRNAs.

The “core circuitry” of pluripotency consists of the homeodomain transcription factors, NANOG, OCT4, and SOX2 in both mouse and human ES cells. These three nuclear factors, closely interacting inside the cell, precisely govern the pluripotent state by regulating a wide range of genes associated with pluripotency signaling networks, including *KLF4*, *C-MYC*, *TBX3*, and *ESRRB* [39], and by influencing the TGF-β and WNT pathways [40]. Several lines of evidence indicated that, in ESCs, small modifications of their complex interplay may affect the pluripotent status: *Sox2* expression oscillations, for example, induced multilineage differentiation [41] while blockades of *Nanog* expression increased their capacity for differentiation into primitive ectoderm cells [42].

The complex C-KIT ligand/C-KIT receptor promoted self-renewal and proliferation in different types of SCs (e.g., embryonal, hemopoietic, and neural SCs) [43].

DNA methylation at the CG dinucleotide in the promoter region of a gene is an epigenetic modification generally associated with gene silencing [44]. Several laboratories have described the epigenetic profile of these pluripotency markers in ESC and iPSCs [5,45], demonstrating that they are hypomethylated in ESCs and that their methylation increases when ESCs begin to differentiate [46]. hiPSCs are similar to ESCs in terms of global chromatin configuration and transcription [5]. Little research is instead available on the epigenetic features of the stemness triad and *C-KIT* in fetal annex-derived SCs. Our study demonstrates that as compared to hiPSCs, *SOX2* and *C-KIT* promoters were hypermethylated in all perinatal SCs, whereas *NANOG* was hypermethylated only in hAFSCs and hAECs. The *OCT4* methylation level was comparable in hiPSCs and perinatal SCs, and the *NANOG* methylation profile was similar in hiPSCs and hFM-MSCs. This observation suggests that placenta derived SCs share some epigenetic features with the pluripotent SCs. Despite this epigenetic similarity, the gene and protein expression of OCT4, SOX2, and NANOG differ among the various SC populations, suggesting that other mechanisms are implicated in the transcriptional regulation of these stemness genes such as histone modifications and non-CpG methylation, but further investigations are needed.

Data from flow cytometry and immunofluorescence demonstrated that the protein expression of the pluripotent markers was significantly lower in perinatal SCs relative to iPSCs. For this reason, we also analyzed the expression of miR-299-3p, miR150-5p, and miR145-5p, which are known to be regulators of NANOG, OCT4, and SOX2 [30,31,32,33]. Our results demonstrated that perinatal SCs generally expressed higher levels of miRNAs known to interfere with the translation of the triad of pluripotency proteins. Although we did not find a statistical correlation, the trend of expression of miR-145-5p was found to be inverse to that of the NANOG, OCT4, and SOX2 proteins. Thus, these data suggest that this miRNA might be involved in the control of the translation of the core of the self-renewal network, but further functional experiments are needed to specifically address this question.

The transcription factors NANOG, OCT4, and SOX2 after the synthesis may migrate into the nucleus to regulate the downstream transcriptional activity of genes related to the pluripotent status. Their subcellular localization in the nuclear or cytoplasmic compartments can be affected by several cellular and molecular elements or by the cell progression in the stemness hierarchy [47]. SOX2 plays its fundamental role in the maintenance of the pluripotency acting predominately at the nucleus level, as demonstrated by its preferential nuclear localization in hiPSCs [48]. OCT4 is present in the nucleus of the ESCs and in the cytoplasm of downstream cells, such as hematopoietic SCs. This evidence suggests that OCT4 localization may be a hierarchic indicator of the stemness [47]. NANOG has been shown to be localized to both the nucleus and cytoplasm, and its functionalities may depend on its subcellular localization. In our experiments, NANOG, OCT4, and SOX2 were clearly detectable, even if at different extents, in the nuclei of the different perinatal SCs. As the nuclear localization of these transcription factors represents an indicator of stemness, this finding suggests that perinatal SCs may be considered early SCs.

Mammalian telomeres are heterochromatic structures at the end of the chromosomes, consisting of repeats of the TTAGGG sequence, which provide chromosomal stability. Somatic cells are characterized by low levels of telomerase, a reverse transcriptase that extends the telomeric repeats, so that the telomere length progressively shortens with cell divisions and triggers cellular senescence. This progressive telomere shortening is one of the molecular mechanisms that underlie ageing, as critically short telomeres trigger chromosome senescence and loss of cell viability. The telomerase activity and the telomere length of the various SC populations are thus important biological characteristics that must be taken into account in cell-based therapeutic strategies. In ESCs telomerase maintains the telomere length and, as a consequence, their proliferative potential; however, in the majority of SCs, the telomerase activity is low regardless of their proliferative capacity, and telomere shortening occurs. Accordingly, our data demonstrated that the telomere length of the perinatal SCs was significantly shorter than that of hiPSCs. Among the various placental derived SCs, the hAFSCs were characterized by significantly longer telomeres.

Telomere length correlated with the developmental pluripotency of ESCs and hiPSCs [49]. ESCs with short telomeres showed a reduction of their teratoma formation capacity and chimera production; moreover, NANOG was expressed at low levels in hESCs with short telomeres, whereas OCT4 and SOX2 expression did not differ in relation to telomere length [50]. According to these data, our study showed that hFM-MSCs have shorter telomeres than hiPSCs and hAFSCs and also lower levels of NANOG expression. Differently, hAECs, although they express high levels of NANOG, are characterized by short telomeres in comparison to hiPSCs and hAFSCs. This is likely the reason for their poor proliferation rate; indeed hAECs can be maintained in culture only for five to six passages [51].

Cellular metabolism describes the biochemical phenotype of the cell. Increasing evidence has demonstrated that the maintenance of stemness and the generation of differentiated lineages are conditioned by cellular metabolism; in particular, a proper quality control of mitochondrial function represents a key factor in SC maintenance and commitment by regulating the redox state [35,36,52]. The bioreduction of water-soluble tetrazolium salts in the MTT assay is generally regarded as an indicator of the cell “redox activity” of the mitochondrial enzymes and electron carriers. Our data demonstrated that perinatal stem cell population analyzed had a comparable, or even higher, “redox activity” to hiPSCs, suggesting that fetal annexes-derived cells, always consider multipotent, might have metabolic features similar to the pluripotent stem cells.

## 5. Conclusions

In conclusion, we found that in perinatal SCs, the pluripotent markers *NANOG, OCT4,* and *SOX2* were not fully silenced, as their promoters were only partially methylated and their expression was regulated at the post transcriptional levels; therefore, the potential of fetal annex-derived SCs might be not restricted to the mesenchymal lineage. While the differentiation properties of the perinatal SCs could be enhanced through a fine-tuned manipulation, the telomere shortening might be the factor limiting their clinical applications in regenerative medicine.

## Figures and Tables

**Table 1:** Cell media and supplements.

Cell Type	Medium	Supplements	Provider
hiPSCs	DMEM F/12	20%KO	All the media and supplements were purchased by Thermo Fisher Scientific (Waltham, MA, USA)
		5 ng/mL bFGF	
		1%MEM non essential amino acid	
hAFSCs	IMDM	20% FBS	
		5 ng/mL bFGF	
hFM-MSCs	DMEM	10% FBS	
hAECs	DMEM	10% FBS	
		10 ng/mL EGF	

All the media were always completed with 1% penicillin/streptomycin, 2 mM l-glutamine.

**Table 2:** The primers for the stem and loop reverse transcription (RT) protocol.

miRNA	Sequence (5′–3′)
miR_299-3p_RT	GTCGTATCCAGTGCAGGGTCCGAGGTATTCGCACTGGATACGACAAGCGG
miR_145-5p_RT	GTCGTATCCAGTGCAGGGTCCGAGGTATTCGCACTGGATACGACAGGGAT
miR_150-5p_RT	GTCGTATCCAGTGCAGGGTCCGAGGTATTCGCACTGGATACGACCACTGG
miR_U6_RT	GAACGCTTCACGAATTTGCGTGTCAT

**Table 3:** The primers for real time PCR.

Gene	Sequence (5′–3′)
*NANOG*-FW	CCAGACCCAGAACATCCAGTC
*NANOG*-RW	CACTGGCAGGAGAATTTGGC
Endo-*OCT4*-FW [18]	GGGTTTTTGGGATTAAGTTCTTCA
Endo-*OCT4*-RW [18]	GCCCCCACCCTTTGTGTT
Endo-*SOX2*-FW [18]	CAAAAATGGCCATGCAGGTT
Endo-*SOX2*-RW [18]	AGTTGGGATCGAACAAAAGCTATT
*c-kit*-FW	CCACACCCTGTTCACTCCTT
c-kit-RW	TTCTGGGAAACTCCCATTTGTG
18S-FW [19]	CATGGCCGTTCTTAGTTGGT
18S-RW [19]	CGCTGAGCCAGTCAGTGTAG
miR-299-3p_FW	CGTGGAGTATGTGGGATGGTAAA
mir-150-5p_FW	GCATGTCTCCCAACCCTTGTA
miR-145-5p_FW	GAAGGTCCAGTTTTCCCAGGA
miR_universal_RW	GTGCAGGGTCCGAGGT
miR_U6_FW	CTCGCTTCGGCAGCACA
miR_U6_RW	AACGCTTCACGAATTTGCGT

**Table 4:** Primer for pyrosequencing.

Gene Promoter	FW Primer	RW Primer	Sequence Primer (5′–3′)	No. of CpGs Assayed	Amplicon Size (bp)
*NANOG*	[Bio]TGTATTTTTAGTAGAGAGGGGGTTT	ACCCAACAACAAATACTTCTAAATTCACC	ATTCACCACCTTTCCAACTT	6	237
*OCT4*	[Bio]ATGGGGGAATTTTTTATATTTTAGAGTT	CACCACCATTAAACAAACATCC	AAAAAATTAAATAATCCCTT	10	373
*SOX2*	AGTAAGGAAGGTTTTGAGGATAGA	[Bio]ATATCATTATTCTCCCCCTCATCCACAA	AGGTTTGGGTTTTTTAAT	6	187
*C-KIT*	GGAGGGGGGAAAAAGTGTATGAAAATTTG	[Bio]TTCTACTCAATTTCTCCACCTACTT	AAATTTGGGTTTTTAGAGTAA	8	179

[Bio] = Biotinylated at 5′ end.
